# Supplementary material for: AIRRscape: An interactive tool for exploring B-cell receptor repertoires and antibody responses
Source: PLoS Comput Biol. 2022 Sep 20;18(9):e1010052. doi: 10.1371/journal.pcbi.1010052 (PMC9524643; doi:10.1371/journal.pcbi.1010052)
Supplement: S1 Data — Fig A in S1 Data. SARS-CoV-2 convergent clonotypes to mAb C102 in the 3_4_11 bin. An 80% identity threshold is used to calculate convergence. Tips are colored by dataset source. Purple tips are published anti-COVID-19 antibodies from 12 different studies, dark gray tips are antibody sequences from a healthy donor BCR repertoire, and orange through brown shaded tips are antibody sequences from COVID-19 patient BCR repertoires. Fig B in S1 Data. SARS-CoV-2 convergent clonotypes to mAb C125 in the 1_3_16 bin. An 80% identity threshold is used to calculate convergence. Tips are colored by dataset source. Purple tips are published anti-COVID-19 antibodies from 7 different studies, dark gray tips are antibody sequences from a healthy donor BCR repertoire, and orange through brown shaded tips are antibody sequences from COVID-19 patient BCR repertoires. Fig C in S1 Data. SARS-CoV-2 convergent clonotypes to mAb BD-494 in the 3_6_11 bin. An 80% identity threshold is used to calculate convergence. Tips are colored by dataset source. Purple tips are published anti-COVID-19 antibodies from 15 different studies, dark gray tips are antibody sequences from a healthy donor BCR repertoire, and orange through brown shaded tips are antibody sequences from COVID-19 patient BCR repertoires. Fig D in S1 Data. AIRRscape heatmaps comparing anti-HIV-1 antibodies and bulk BCR repertoires of eight HIV-1 patients. Fig E in S1 Data. HIV-1 convergent clonotypes to mAb CAP351_6m_6041 (Setliff et al. 2018; Fig 3) in the 1_6_14 bin. A 70% identity threshold is used to calculate convergence. Tips are colored by dataset source. Purple tips are published anti-COVID-19 antibodies, and green shaded tips are antibody sequences from HIV-1 patient BCR repertoires. Fig F in S1 Data. AIRRscape heatmaps comparing isolated dengue plasmablasts and bulk BCR repertoires of dengue patients from Colombian and Nicaraguan cohorts. Fig G in S1 Data. Dengue convergent clonotypes to CF6 (Zanini et al. 2018). An 80% iden [file pcbi.1010052.s001.pdf]

AIRRscape: an interactive tool for exploring B-cell receptor repertoires and antibody responses

Eric Waltari[1,*], Saba Nafees[1], Krista M. McCutcheon[1,#], Joan Wong[1], John E. Pak[1,*]

[1]Chan Zuckerberg Biohub, San Francisco, California, United States of America

[*]Corresponding authors

E-mail: eric.waltari@czbiohub.org (EW) or john.pak@czbiohub.org (JEP)

[#]Current address: Immune-Onc Therapeutics Inc, Palo Alto, California, United States of America

Short title (65 characters): *AIRRscape: an interactive tool for exploring antibody repertoires*

Keywords: AIRR, antibodies, B cell repertoire, convergent antibody response, data visualization, immune repertoire

Supporting Information

Fig A in S1 Data. SARS-CoV-2 convergent clonotypes to mAb C102 in the 3_4_11 bin. An 80% identity threshold is used to calculate convergence. Tips are colored by dataset source. Purple tips are published anti-COVID-19 antibodies from 12 different studies, dark gray tips are antibody sequences from a healthy donor BCR repertoire, and orange through brown shaded tips are antibody sequences from COVID-19 patient BCR repertoires.

Fig B in S1 Data. SARS-CoV-2 convergent clonotypes to mAb C125 in the 1_3_16 bin. An 80% identity threshold is used to calculate convergence. Tips are colored by dataset source. Purple tips are published anti-COVID-19 antibodies from 7 different studies, dark gray tips are antibody sequences from a healthy donor BCR repertoire, and orange through brown shaded tips are antibody sequences from COVID-19 patient BCR repertoires.

Fig C in S1 Data. SARS-CoV-2 convergent clonotypes to mAb BD-494 in the 3_6_11 bin. An 80% identity threshold is used to calculate convergence. Tips are colored by dataset source. Purple tips are published anti-COVID-19 antibodies from 15 different studies, dark gray tips are antibody sequences from a healthy donor BCR repertoire, and orange through brown shaded tips are antibody sequences from COVID-19 patient BCR repertoires.

Fig D in S1 Data. AIRRscape heatmaps comparing anti-HIV-1 antibodies and bulk BCR repertoires of eight HIV-1 patients.

Fig E in S1 Data. HIV-1 convergent clonotypes to mAb CAP351_6m_6041 (Setliff et al. 2018; Fig 3) in the 1_6_14 bin. A 70% identity threshold is used to calculate convergence. Tips are colored by dataset source. Purple tips are published anti-COVID-19 antibodies, and green shaded tips are antibody sequences from HIV-1 patient BCR repertoires.

Fig F in S1 Data. AIRRscape heatmaps comparing isolated dengue plasmablasts and bulk BCR repertoires of dengue patients from Colombian and Nicaraguan cohorts.

Fig G in S1 Data. Dengue convergent clonotypes to CF6 (Zanini et al. 2018). An 80% identity threshold is used to calculate convergence. Tips are colored by dataset source. Purple tips are plasmablast sequences reported by Zanini et al. (2018) isolated from two Colombian patients (d13 and d20), blue tips are antibody sequences from the BCR repertoire of patient d13, and gold tips are antibody sequences from a cohort of Nicaraguan patient BCR repertoires.

Fig H in S1 Data. Dengue convergent clonotypes to CF7 (Zanini et al. 2018). An 80% identity threshold is used to calculate convergence. Tips are colored by dataset source. Purple tips are plasmablast sequences reported by Zanini et al. (2018) isolated from two Colombian patients (d13 and d20), blue tips are antibody sequences from the BCR repertoire of patient d13, and gold tips are antibody sequences from a cohort of Nicaraguan patient BCR repertoires.

Fig I in S1 Data. Dengue convergent clonotypes to Parameswaran et al. (2018) motif ARQIGNWFDP similar to CF1 (Zanini et al. 2018). An 80% identity threshold is used to calculate convergence. Tips are colored by dataset source. Purple tips are plasmablast sequences reported by Zanini et al. (2018) isolated from two Colombian patients (d13 and d20),

blue tips are antibody sequences from the BCR repertoire of patient d13, and gold tips are antibody sequences from a cohort of Nicaraguan patient BCR repertoires.

Fig J in S1 Data. SARS-CoV-2, HIV-1, & dengue convergent clonotypes to anti-SARS-CoV-2 mAb C102 in the 3_4_11 bin. An 80% identity threshold is used to calculate convergence. Tips are colored by dataset source. Purple tips are published anti-COVID-19 antibodies from 12 different studies, dark gray tips are antibody sequences from a healthy donor BCR repertoire, and orange through brown shaded tips are antibody sequences from COVID-19 patient BCR repertoires. Green shaded tips are antibody sequences from HIV-1 patient BCR repertoires. Gold tips are antibody sequences from a cohort of Nicaraguan dengue patient BCR repertoires.

Fig K in S1 Data. SARS-CoV-2, HIV-1, & dengue convergent clonotypes to anti-SARS-CoV-2 mAb BD-494 in the 3_6_11 bin. An 80% identity threshold is used to calculate convergence. Tips are colored by dataset source. Purple tips are published anti-COVID-19 antibodies from 15 different studies, dark gray tips are antibody sequences from a healthy donor BCR repertoire, and orange through brown shaded tips are antibody sequences from COVID-19 patient BCR repertoires. Green shaded tips are antibody sequences from HIV-1 patient BCR repertoires. Gold tips are antibody sequences from a cohort of Nicaraguan dengue patient BCR repertoires.

Fig L in S1 Data. HIV-1 & dengue convergent clonotypes to anti-HIV mAb 02-o (Setliff et al. 2018; Fig. 4) in the 1_4_13 bin. An 80% identity threshold is used to calculate convergence. Tips are colored by dataset source. Purple tips are published anti-HIV-1 antibodies, while green shaded tips are antibody sequences from HIV-1 patient BCR repertoires. Blue tips are antibody sequences from the BCR repertoire of dengue patient d13, and gold tips are antibody sequences from a cohort of Nicaraguan dengue patient BCR repertoires.

Fig M in S1 Data. SARS-CoV-2 & dengue convergent clonotypes to anti-dengue mAb CF6 (Zanini et al. 2018) in the 1_4_13 bin. An 80% identity threshold is used to calculate convergence. Tips are colored by dataset source. Purple tips are plasmablast sequences reported by Zanini et al. (2018) isolated from two Colombian dengue patients (d13 and d20), blue tips are antibody sequences from the BCR repertoire of dengue patient d13, and gold tips are antibody sequences from a cohort of Nicaraguan dengue patient BCR repertoires. Brown shaded tips are antibody sequences from COVID-19 patient BCR repertoires.

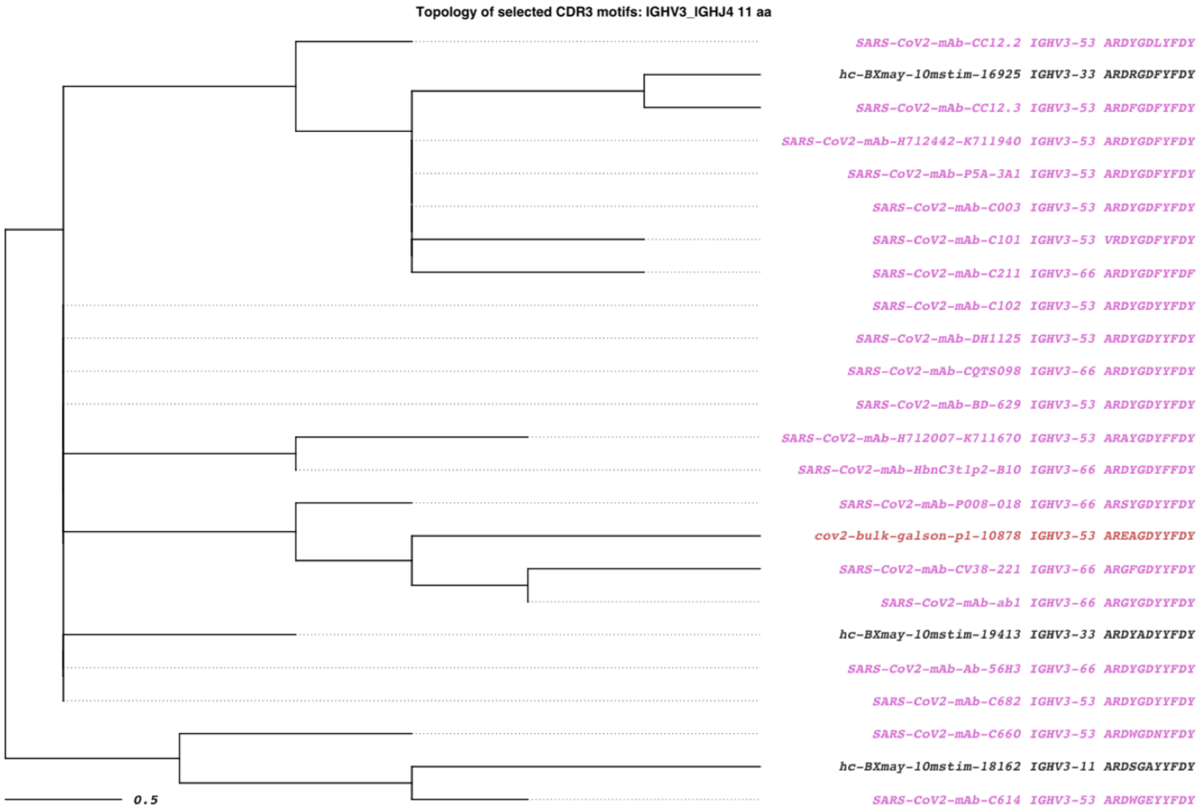

Fig A in S1 Data. SARS-CoV-2 convergent clonotypes to mAb C102 in the 3_4_11 bin. An 80% identity threshold is used to calculate convergence. Tips are colored by dataset source. Purple tips are published anti-COVID-19 antibodies from 12 different studies, dark gray tips are antibody sequences from a healthy donor BCR repertoire, and orange through brown shaded tips are antibody sequences from COVID-19 patient BCR repertoires.

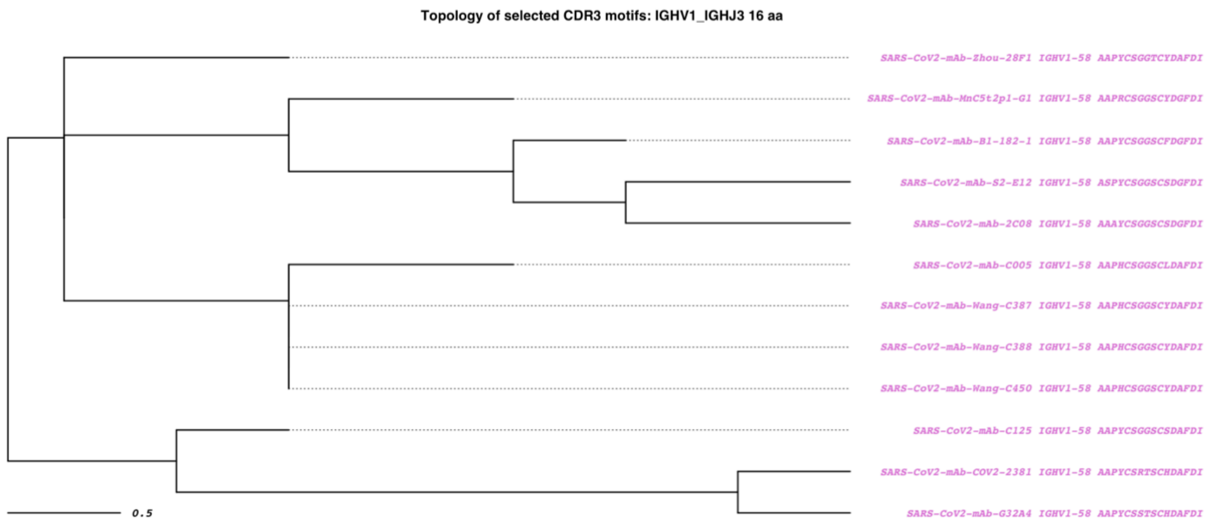

Fig B in S1 Data. SARS-CoV-2 convergent clonotypes to mAb C125 in the 1_3_16 bin. An 80% identity threshold is used to calculate convergence. Tips are colored by dataset source. Purple tips are published anti-COVID-19 antibodies from 7 different studies, dark gray tips are antibody sequences from a healthy donor BCR repertoire, and orange through brown shaded tips are antibody sequences from COVID-19 patient BCR repertoires.

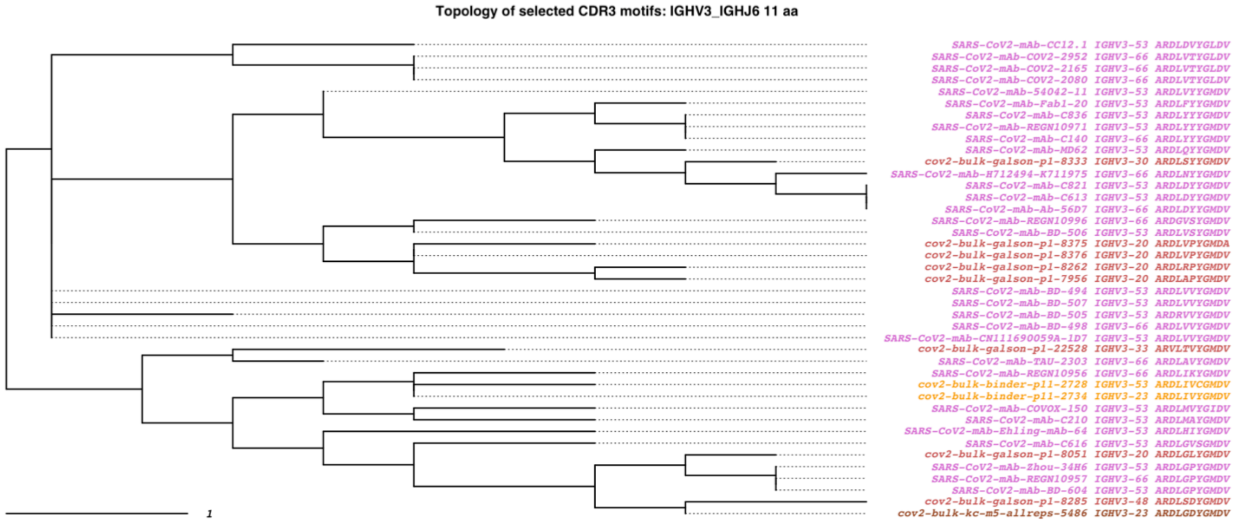

Fig C in S1 Data. SARS-CoV-2 convergent clonotypes to mAb BD-494 in the 3_6_11 bin. An 80% identity threshold is used to calculate convergence. Tips are colored by dataset source. Purple tips are published anti-COVID-19 antibodies from 15 different studies, dark gray tips are antibody sequences from a healthy donor BCR repertoire, and orange through brown shaded tips are antibody sequences from COVID-19 patient BCR repertoires.

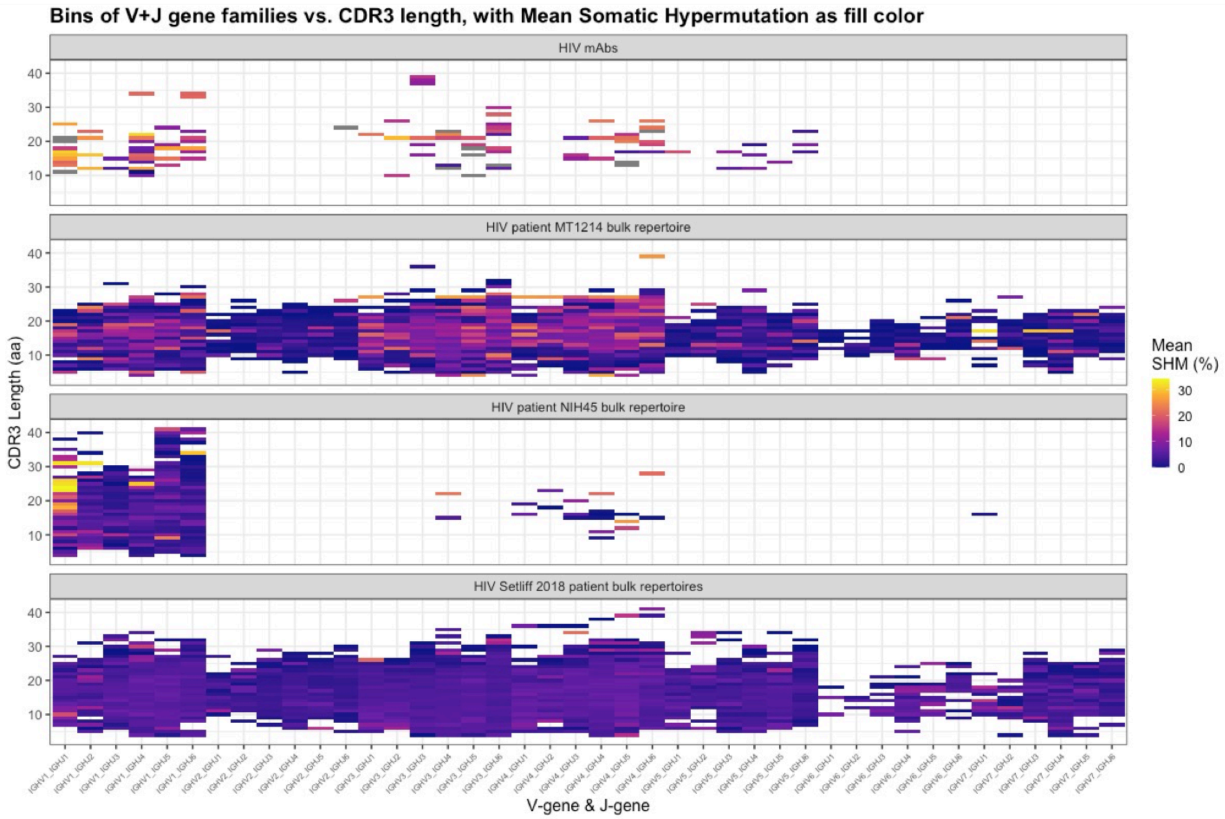

Fig D in S1 Data. AIRRscape heatmaps comparing anti-HIV-1 antibodies and bulk BCR repertoires of eight HIV-1 patients.

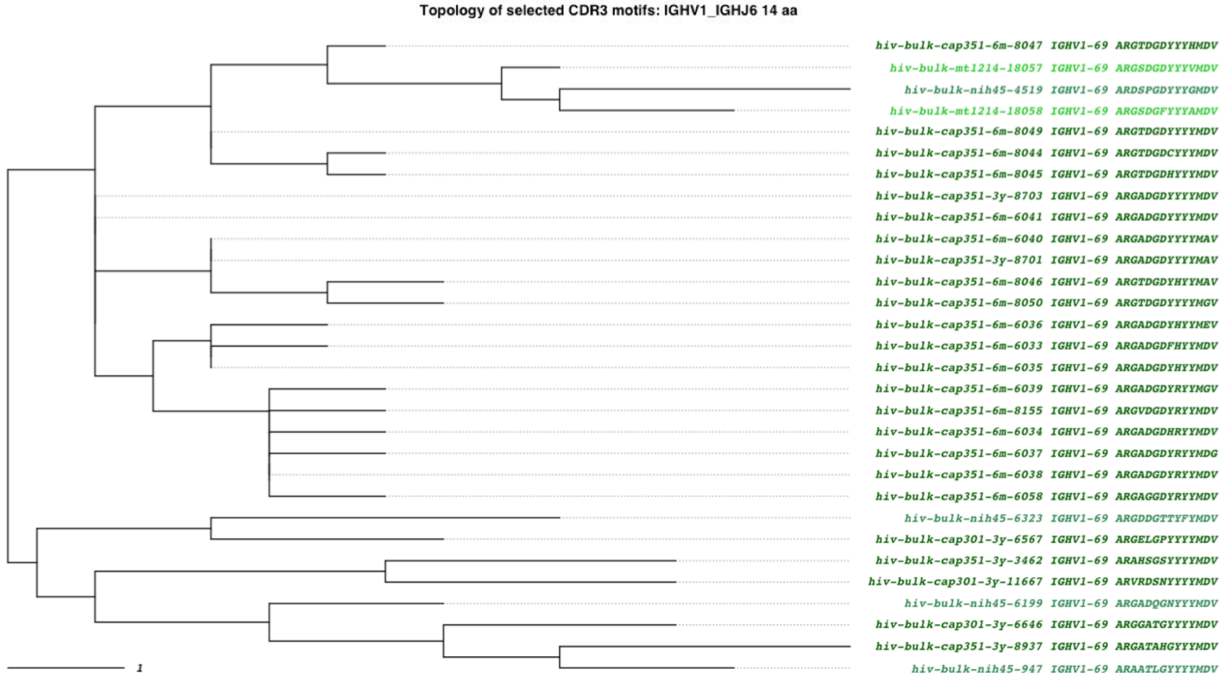

Fig E in S1 Data. HIV-1 convergent clonotypes to mAb CAP351_6m_6041 (Setliff et al. 2018; Fig 3) in the 1_6_14 bin. A 70% identity threshold is used to calculate convergence. Tips are colored by dataset source. Green shaded tips are antibody sequences from HIV-1 patient BCR repertoires.

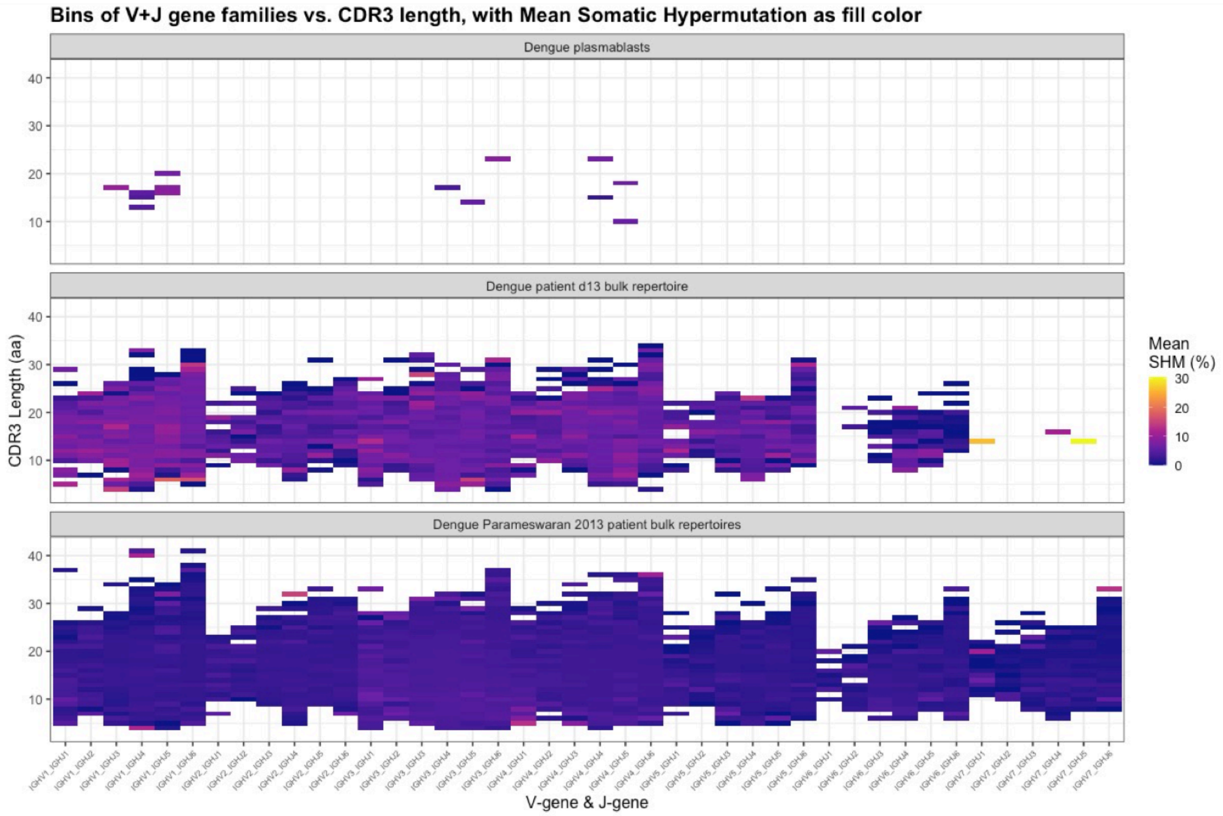

Fig F in S1 Data. AIRRscape heatmaps comparing isolated dengue plasmablasts and bulk BCR repertoires of dengue patients from Colombian and Nicaraguan cohorts.

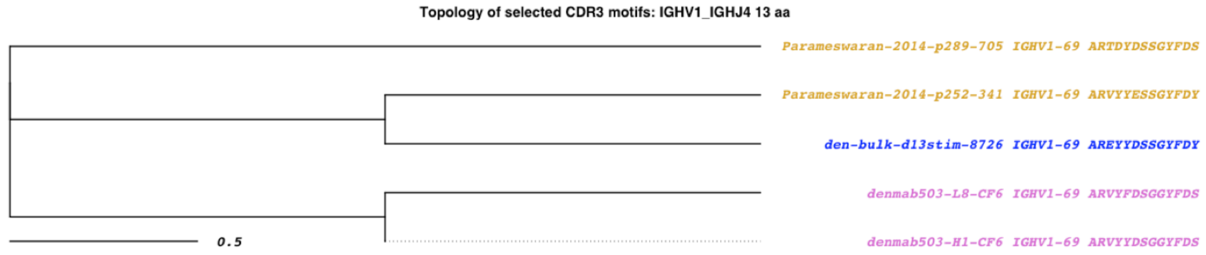

Fig G in S1 Data. Dengue convergent clonotypes to CF6 (Zanini et al. 2018). An 80% identity threshold is used to calculate convergence. Tips are colored by dataset source. Purple tips are plasmablast sequences reported by Zanini et al. (2018) isolated from two Colombian patients (d13 and d20), blue tips are antibody sequences from the BCR repertoire of patient d13, and gold tips are antibody sequences from a cohort of Nicaraguan patient BCR repertoires.

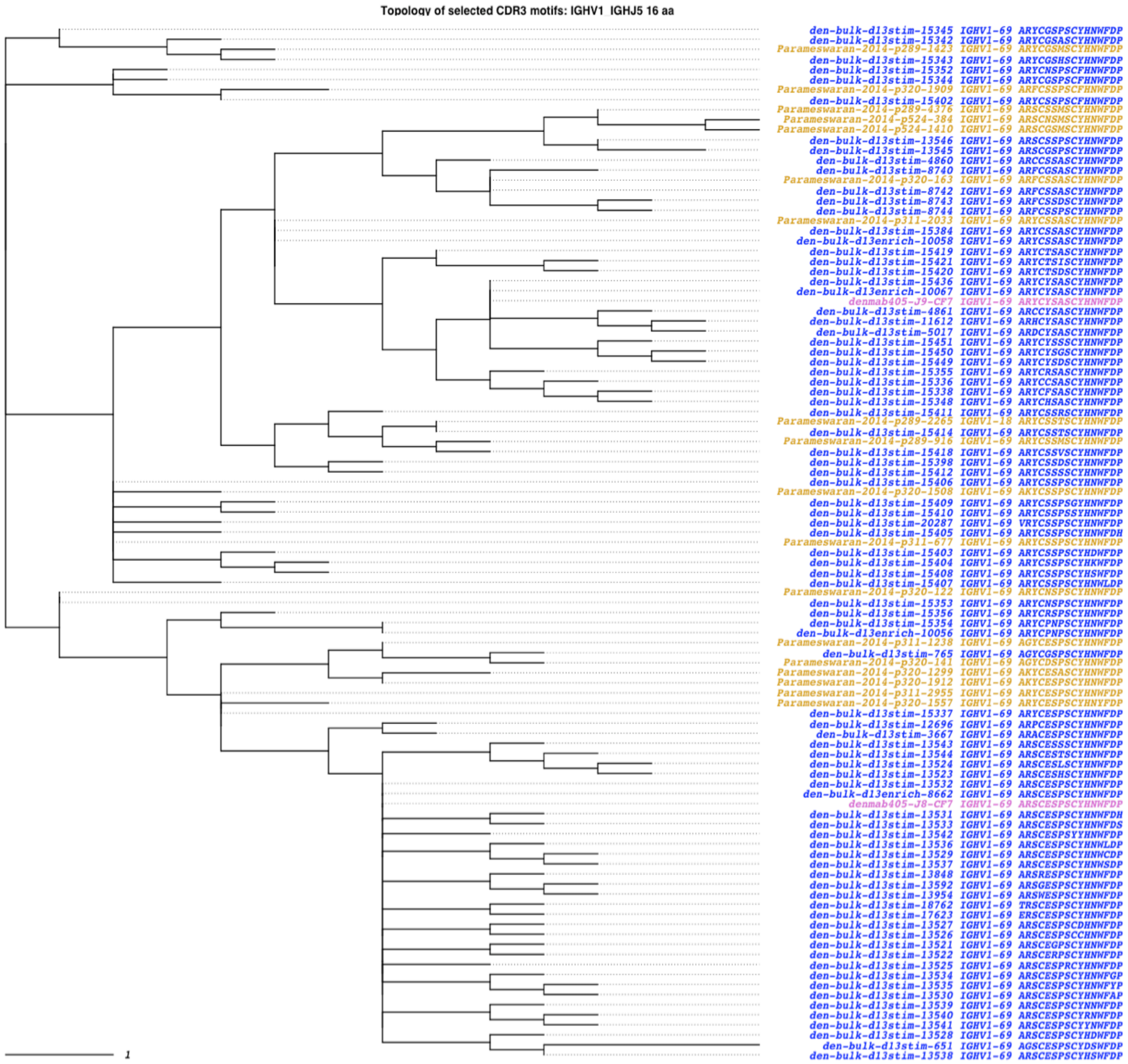

Fig H in S1 Data. Dengue convergent clonotypes to CF7 (Zanini et al. 2018). An 80% identity threshold is used to calculate convergence. Tips are colored by dataset source. Purple tips are plasmablast sequences reported by Zanini et al. (2018) isolated from two Colombian patients (d13 and d20), blue tips are antibody sequences from the BCR repertoire of patient d13, and gold tips are antibody sequences from a cohort of Nicaraguan patient BCR repertoires.

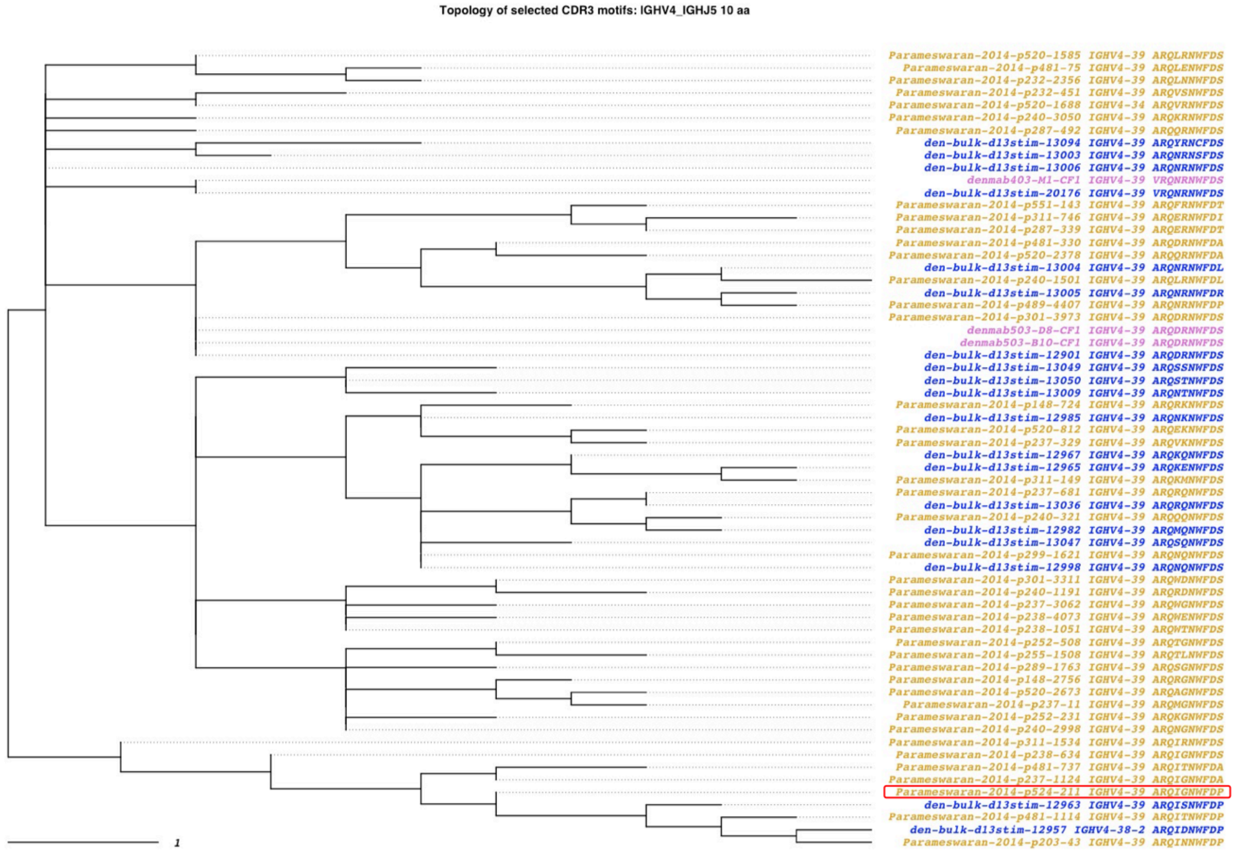

Fig I in S1 Data. Dengue convergent clonotypes to Parameswaran et al. (2018) motif ARQIGNWFDP similar to CF1 (Zanini et al. 2018). An 80% identity threshold is used to calculate convergence. Tips are colored by dataset source. Purple tips are plasmablast sequences reported by Zanini et al. (2018) isolated from two Colombian patients (d13 and d20), blue tips are antibody sequences from the BCR repertoire of patient d13, and gold tips are antibody sequences from a cohort of Nicaraguan patient BCR repertoires.

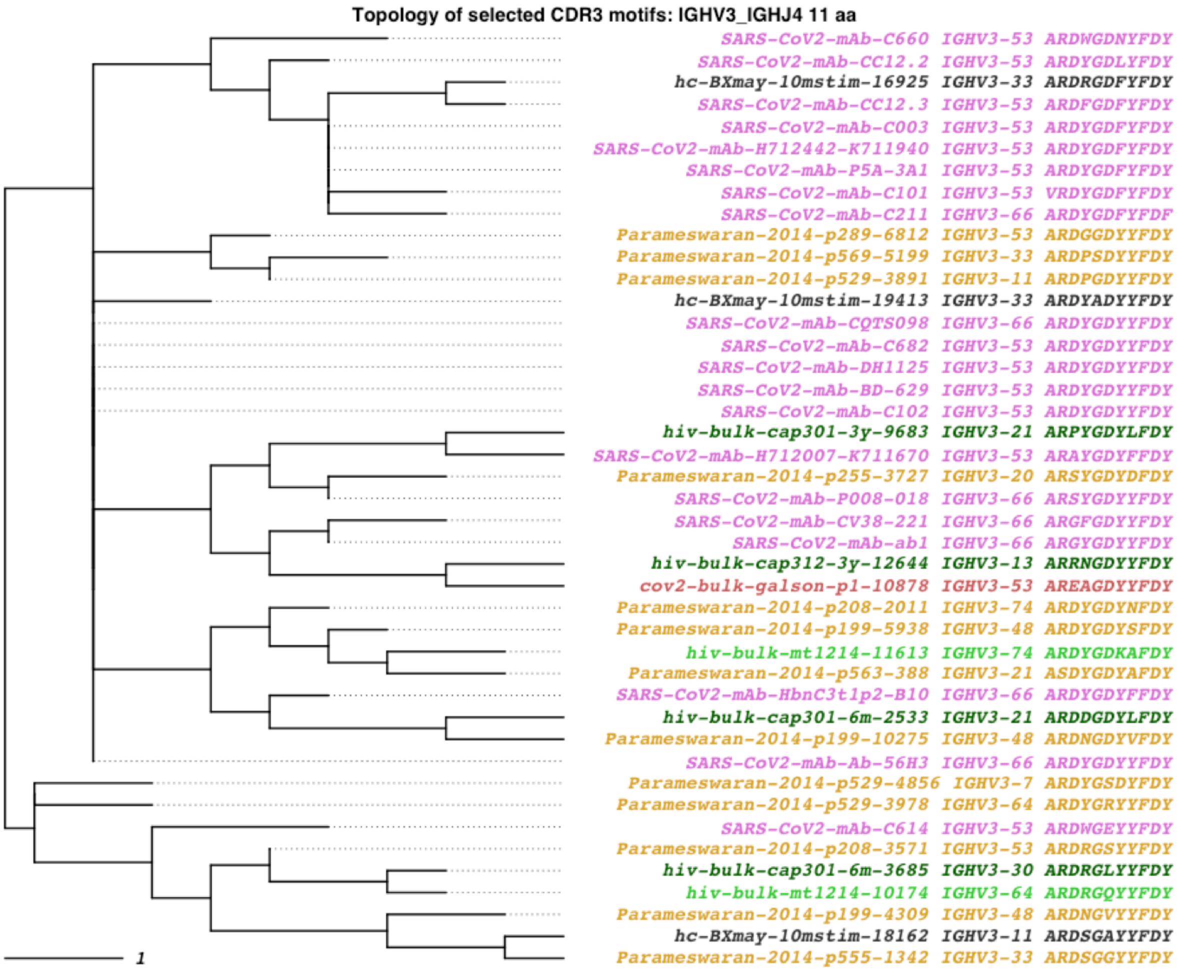

Fig J in S1 Data. SARS-CoV-2, HIV-1, & dengue convergent clonotypes to anti-SARS-CoV-2 mAb C102 in the 3_4_11 bin. An 80% identity threshold is used to calculate convergence. Tips are colored by dataset source. Purple tips are published anti-COVID-19 antibodies from 12 different studies, dark gray tips are antibody sequences from a healthy donor BCR repertoire, and orange through brown shaded tips are antibody sequences from COVID-19 patient BCR repertoires. Green shaded tips are antibody sequences from HIV-1 patient BCR repertoires. Gold tips are antibody sequences from a cohort of Nicaraguan dengue patient BCR repertoires.

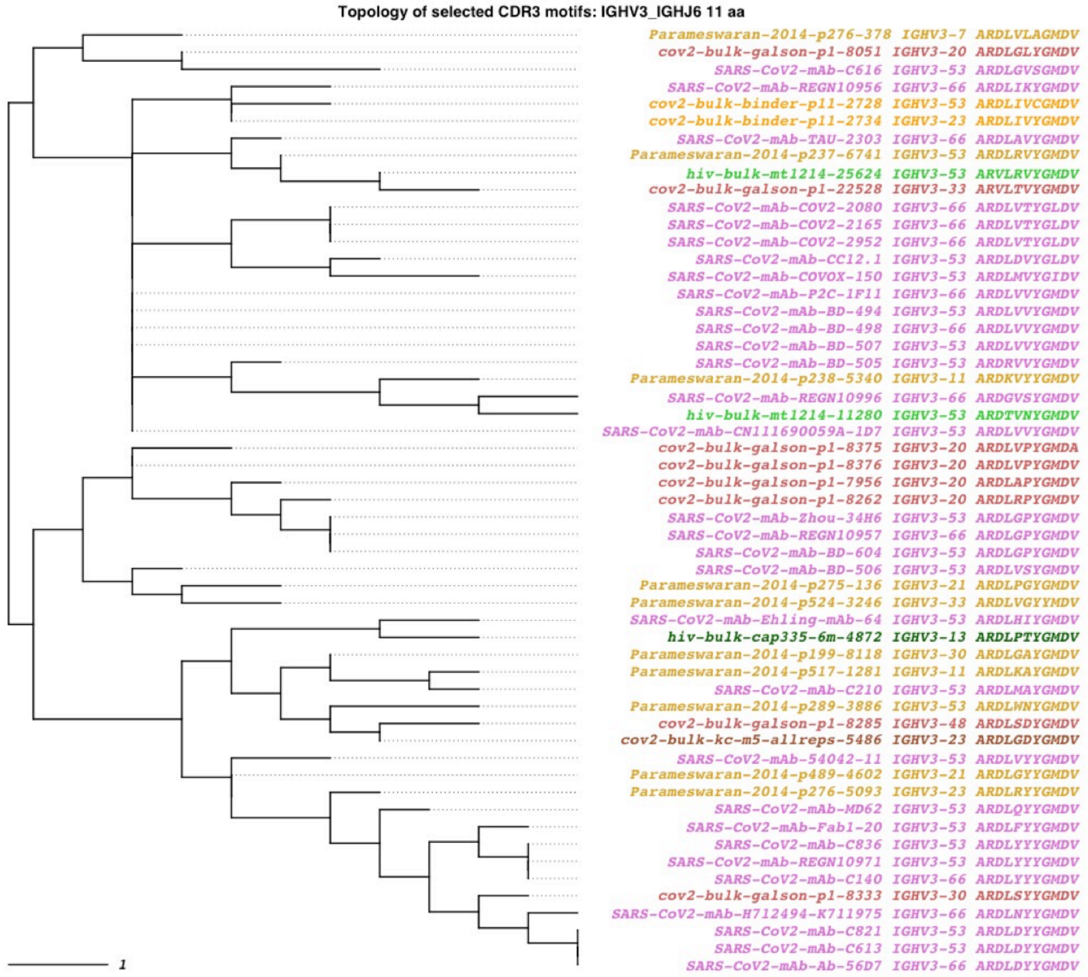

Fig K in S1 Data. SARS-CoV-2, HIV-1, & dengue convergent clonotypes to anti-SARS-CoV-2 mAb BD-494 in the 3_6_11 bin. An 80% identity threshold is used to calculate convergence. Tips are colored by dataset source. Purple tips are published anti-COVID-19 antibodies from 15 different studies, dark gray tips are antibody sequences from a healthy donor BCR repertoire, and orange through brown shaded tips are antibody sequences from COVID-19 patient BCR repertoires. Green shaded tips are antibody sequences from HIV-1 patient BCR repertoires. Gold tips are antibody sequences from a cohort of Nicaraguan dengue patient BCR repertoires.

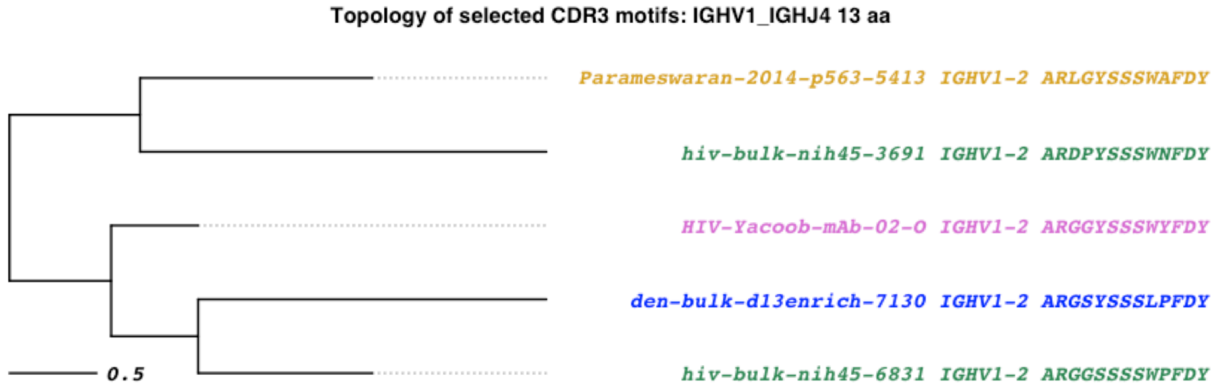

Fig L in S1 Data. HIV-1 & dengue convergent clonotypes to anti-HIV mAb 02-o (Setliff et al. 2018; Fig. 4) in the 1_4_13 bin. An 80% identity threshold is used to calculate convergence. Tips are colored by dataset source. Purple tips are published anti-HIV-1 antibodies, while green shaded tips are antibody sequences from HIV-1 patient BCR repertoires. Blue tips are antibody sequences from the BCR repertoire of dengue patient d13, and gold tips are antibody sequences from a cohort of Nicaraguan dengue patient BCR repertoires.

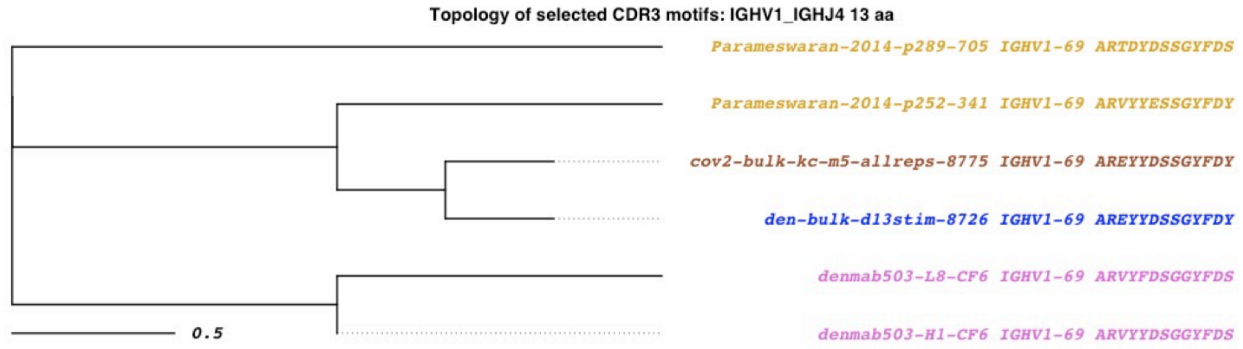

Fig M in S1 Data. SARS-CoV-2 & dengue convergent clonotypes to anti-dengue mAb CF6 (Zanini et al. 2018) in the 1_4_13 bin. An 80% identity threshold is used to calculate convergence. Tips are colored by dataset source. Purple tips are plasmablast sequences reported by Zanini et al. (2018) isolated from two Colombian dengue patients (d13 and d20), blue tips are antibody sequences from the BCR repertoire of dengue patient d13, and gold tips are antibody sequences from a cohort of Nicaraguan dengue patient BCR repertoires. Brown shaded tips are antibody sequences from COVID-19 patient BCR repertoires.
