# Supplementary material for: AIRRscape: An interactive tool for exploring B-cell receptor repertoires and antibody responses
Source: PLoS Comput Biol. 2022 Sep 20;18(9):e1010052. doi: 10.1371/journal.pcbi.1010052 (PMC9524643; doi:10.1371/journal.pcbi.1010052)
Supplement: Attachment — Submitted filename: airrscape review.docx [file pcbi.1010052.s002.docx]

The authors describe AIRRscape: an interactive tool for the exploration of receptor repertoires. The tool focuses on the exploration of shared or near-identical CDR3 sequences between repertoires. Source code is published under the MIT open source license.

As the use of AIRR-seq grows, there is a growing need for tools that are easy to use and can provide useful insights into repertoire characteristics. AIRRscape is such a tool. It takes as input one or more annotated repertoires, requiring little additional processing. The tool does not present sequences clustered into clonal families (but see note below on line 105), instead binning sequences by CDR3 length, V family, and J gene.

Overall, the tool would be useful for initial analysis of small numbers of repertoires, particularly where the user is interested to identify similar CDR3 sequences occurring in multiple repertoires, or to look for near-matches to motifs of known antibodies of interest. There are, however, some key points which should be addressed to ensure that users understand the limitations of analysis.

Key Points

The application begins by displaying a heatmap in which sequences from each repertoire are binned by V family/J gene on one axis, and CDR3 length on the other. The heatmap can be coloured by mean SHM or sequence count. While this provides a useful visual map from which to drill down into bins, the categorization is too broad to see meaningful differentiation between the repertoires presented, except for the overall level of SHM in some samples which could easily be expressed as a metric. With the current level of granularity, and in the absence of metrics, statements such as “the overall pattern … does not differ” (line 210) say more about the tool than the repertoires. Enhancement of this screen and the introduction of metrics are opportunities for future development, but, at the current level of development, comparison between repertoires should be restricted to specific standout points and broad statements made on the basis of visual assessment should be avoided.

The repertoire read count varies widely between samples. The samples with smaller read counts are likely to have thinner vertical bands as a result of the lower numbers. This could be addressed, for example, by providing a sub-sampling option. Otherwise, a note should be made regarding sample size bias.

The SHM metric is used widely in the paper. It is calculated as 100 - v_identity and therefore reflects the level of mutation in the V-REGION rather than overall SHM, which would look also at the level of mutation in the junction, and would therefore require clonal analysis. It would be better to use a different name, perhaps V-SHM, to make this clear.

The strength of the tool is in the analysis of convergent sequences, and its utility is usefully shown with reference to the three published studies. However it is important to note that amino acid sequence convergence at the levels analysed is an imperfect predictor of functional convergence: sequences will require confirmatory analysis by other means. Because this point is sometimes overlooked, it should be mentioned in the discussion so that users are aware of it.

Other points

Line 105, 367 – given that clonal analysis is not performed, please explain why changeo-clone needed. Alternatively, if it is needed, why not use the results and incorporate clonal analysis? Do the authors recommend Olmsted over AIRRscape where clonal analysis is available? The comparative merits of the two tools should be set out in greater detail.

Line 221 – ‘more easily and broadly elicited’ – possibly in the narrow context of mutation steps but this is a sweeping statement.

Line 375 – heavy V-gene *family* (which is a very broad metric).

Line 389 – This is potentially an important finding, but the dengue sequences are not represented in the covid trees, and the description of these dengue sequences at line 240 is not clear. The reference to 3.3 is not helpful. Specifically it is important to know whether sequences identical to known functional COVID antibodies were found in the dengue patients - Otherwise the finding needs to be qualified or supported by more evidence that the sequences are functional against SAVS-CoV-2.
