# Supplementary material for: AIRRscape: An interactive tool for exploring B-cell receptor repertoires and antibody responses
Source: PLoS Comput Biol. 2022 Sep 20;18(9):e1010052. doi: 10.1371/journal.pcbi.1010052 (PMC9524643; doi:10.1371/journal.pcbi.1010052)
Supplement: Attachment — Submitted filename: AIRRscape.docx [file pcbi.1010052.s003.docx]

# Review: AIRRscape

## Major Points

There is insufficient discussion about previous tools in this space. While there aren't many AIRR-seq visualization tools to compare to, there are some:

- "AncesTree: An interactive immunoglobulin lineage tree visualizer" by Foglierini et al.
- Olmsted by Ralph et al.
- "sumrep: A Summary Statistic Framework for Immune Receptor Repertoire Comparison and Model Validation" by Olson et al.

The above tools don't focus on convergence analysis, like AIRRscape does. Then again, the paper is written as if AIRRscpae is a general tool and not one focused on convergence analysis, an exciting area in need of visualization tools. The authors should update the text to make this clear and better position their tool in the space of the AIRR-seq visualization tools. This would increase its novelty and discoverability.

Since the tool is focused on convergence analysis, it should be much easier to identify bins that contain rearrangements from multiple people and/or from people in multiple groups. Data can be faceted by source, then the user has to bounce back and forth trying to identify equivalent bins. Or the combined data could be used but then the user has to go searching for bins with overlapping data. An option should be added to highlight bins with overlapping sequences.

As a practitioner of BCR analysis, it's not clear from the text how much work is needed to get your own data ready for use in AIRRscape. The paper does mention AIRR community standards but doesn't describe what other user supplied metadata is needed in order to get the faceted displays shown in the paper. Maybe this is simple but it's not described in the paper, even briefly.

The authors commitment to open science, publishing on bioRxiv and making their code fully available on github is to be lauded.

## Minor Points

Some of the author's analysis includes antibodies for which we only know the a.a. sequence. The method they use to reverse translate (using tblastn) is clear. The github repository has some additional details: "search for 100% sequence match to nucleotide sequences". It is not clear how many sequences were reverse translated with 100% a.a. match. Was it all of them? The authors also mention in the github repository that some sequences were "remove… with artificially high SHM due to codon optimization". How many were removed? This suggests that SHM levels for antibodies for which we only know the a.a. sequence should be left as NAs in the data.

gf_vgene should be used for the V-segment gene family instead of the ambiguous gf.

I like that the authors emphasize that the phylogenetic trees don't indicate common ancestry. This is a point that is something forgotten or ignored.

The discussion section spends too much time recapitulating the results section. This should be reduced.
