# Supplementary material for: AIRRscape: An interactive tool for exploring B-cell receptor repertoires and antibody responses
Source: PLoS Comput Biol. 2022 Sep 20;18(9):e1010052. doi: 10.1371/journal.pcbi.1010052 (PMC9524643; doi:10.1371/journal.pcbi.1010052)
Supplement: Attachment — Submitted filename: 2022-AIRRscape- an interactive tool for exploring B-cell receptor repertoires and antibody responses.docx [file pcbi.1010052.s004.docx]

Waltari et al present AIRRscape, an R Shiny tool to visualize and analyze B-cell receptor repertoires. This is performed by collapsing receptor sequences by their V gene, J gene and CDR3 amino acid sequence into a single bin, and calculating the average SHM, max SHM and # of read counts per bin. The authors then show the application of AIRRScape on four different datasets, a COVID-19, HIV-1, Dengue and healthy control datasets. As the authors note, adaptive immune receptor repertoire sequencing (AIRR-seq) is becoming more extensively used and there is a critical need to develop tools to analyze datasets in both an interactive and static fashion. This has been critical in other fields such as genomics with the tools IGV and the web browser application ‘UCSC Genome Browser’. While AIRRscape currently has limitations, such as only considering CDR3, as the authors note, AIRRscape represents an initial stage of development for the visualization and interaction of AIRR-seq data.

Major revisions:

1. It would be good to create a separate R script containing the `shinyprocess` function that accepts an AIRR community standard file and creates a data file needed by AIRRscape. While this might defeat the purpose of being user-friendly to people who do not use the command line, the ‘airrscape_preprocessing.R’ file doesn’t seem user-friendly to individuals with less programming experience. At the very least the GitHub page should have instructions on how to visualize a dataset that is not part of the AIRRscape. Instructions should include what lines to edit, lines to run, etc.
2. In lines 210-212, the authors state that “This analysis revealed that the overall pattern of heavy chain V- gene + J-gene usage versus CDRH3 length distribution does not differ among the CoV-AbDab dataset, the four patient repertoires, and the healthy control repertoire (Fig 4).” While the patterns across datasets look similar, there are clear differences. There seems to be no SARS-CoV2 mAbs with IGHV6 genes and IGHV2_IGHJ1 and IGHV2_IGHJ2 gene pairs. The Nielsen et al cohort also doesn’t seem to have IGHV5_IGHJ1 or IGHV5_J2 sequences. The phrase “does not differ” in line 211 may be too strong. It would be good to run statistical test to determine if that is true. Alternatively, ‘does not differ’ can be loosened up to ‘visually the distributions appear similar’, and the differences can be expanded on.
3. I couldn’t create a topology using the code on GitHub. I kept receiving an ‘Error: argument is of length zero’ message. I tried on firefox and chrome. I had the rows selected with the same CDR3 length but it kept producing the same message.

Minor revisions:

1. Under ‘2.2 Data processing’, can the authors add the parameters and values used for ‘changeo-igblast’? It will also be good to include the allele database used for ‘changeo-igblast’. While AIRRscape is agnostic to igblast parameters and allele datatabases used, this information should be added for completeness and reproducibility sake.
2. A reference to VDJbase is needed. VDJbase produces reports and also has interactive modules that lets individuals analyze publicly available AIRR-seq data and determine haplotype, gene and allele usage and other AIRR-seq metrics.
3. For table 1, ‘Sample’ presumably means the sample used from the dataset for this study. Can the authors explain why some rows in the table don’t have a ‘Sample’ id.
4. For lines 108 to 110, can the authors explain which database was used for tblastn, and why the protein sequences were not converted to nucleotide sequences and aligned to an allele database?
5. There is a field in AIRR standards that contains the CDR3 sequence. Could this be used instead determining the CDR3 sequence from the junction field?
6. In line 262-263, the authors state that the V-gene + J-gene usage is similar. However, gene usage is defined either as clonal gene usage or repertoire-wide gene usage and is the frequency of reads (or clones) with a particular gene. Figure 2A doesn’t show gene usage. It shows the SHM rate for different pairs of IGHV+J genes+CDR3 length sequences. I believe the authors mean the IGHV+J gene pairs are similar. Line 211 also makes reference to V-gene + J-gene usage. This should be changed to ‘pair’
7. R version > 4.1.0 is needed for phangorn, it would be good to add this minimum version to the GitHub page.
