# Supplementary material for: AIRRscape: An interactive tool for exploring B-cell receptor repertoires and antibody responses
Source: PLoS Comput Biol. 2022 Sep 20;18(9):e1010052. doi: 10.1371/journal.pcbi.1010052 (PMC9524643; doi:10.1371/journal.pcbi.1010052)
Supplement: Attachment — Submitted filename: AIRRScape reviews and reponses.docx [file pcbi.1010052.s005.docx]

**Dr. Gur Yaari (Guest Editor) and Dr. Rob De Boer (Deputy Editor)**

**PLOS Computational Biology**

**Dear Dr. Yaari and Dr. De Boer,**

**Thank you for providing the referees’ comments for our manuscript “AIRRscape: an interactive tool for exploring B-cell receptor repertoires and antibody responses”. They were generally positive and made excellent suggestions for improving both the manuscript and the tool. In this resubmission, we have addressed all of their critiques and incorporated new features, which now includes a freely accessible website (**[**https://airrscape.czbiohub.org**](https://airrscape.czbiohub.org)**) that permits users to immediately import and visualize their own AIRR-seq repertoire data or visualize the pre-loaded datasets analyzed in the manuscript. In addition, the updated source code and detailed documentation remains fully available at** [**https://github.com/czbiohub/AIRRscape**](https://github.com/czbiohub/AIRRscape)**.**

**Major additions to the manuscript include new text and figures that examine the convergent motifs between SARS-CoV-2, HIV-1, and dengue datasets, as well as a new Table 1 that summarizes existing open-source tools that are available for antibody repertoire analysis and visualization.**

**We would like to thank the referees for their thorough review of the manuscript. Please find below a point-by-point response to the referee comments.**

**Sincerely,**

**Eric Waltari and John E. Pak**

**Reviewer 1:**

The authors describe AIRRscape: an interactive tool for the exploration of receptor repertoires. The tool focuses on the exploration of shared or near-identical CDR3 sequences between repertoires. Source code is published under the MIT open source license.

As the use of AIRR-seq grows, there is a growing need for tools that are easy to use and can provide useful insights into repertoire characteristics. AIRRscape is such a tool. It takes as input one or more annotated repertoires, requiring little additional processing. The tool does not present sequences clustered into clonal families (but see note below on line 105), instead binning sequences by CDR3 length, V family, and J gene.

**Overall, the tool would be useful for initial analysis of small numbers of repertoires, particularly where the user is interested to identify similar CDR3 sequences occurring in multiple repertoires, or to look for near-matches to motifs of known antibodies of interest.** There are, however, some key points which should be addressed to ensure that users understand the limitations of analysis.

We thank the reviewer for recognizing AIRRscape as a useful bioinformatic tool for immune repertoire analyses and for uncovering convergence of antibody motifs.

**Key Points**

The application begins by displaying a heatmap in which sequences from each repertoire are binned by V family/J gene on one axis, and CDR3 length on the other. The heatmap can be coloured by mean SHM or sequence count. While this provides a useful visual map from which to drill down into bins, the categorization is too broad to see meaningful differentiation between the repertoires presented, except for the overall level of SHM in some samples which could easily be expressed as a metric. With the current level of granularity, and in the absence of metrics, statements such as “the overall pattern … does not differ” (**line 210**) say more about the tool than the repertoires. Enhancement of this screen and the introduction of metrics are opportunities for future development, but, at the current level of development, comparison between **repertoires should be restricted to specific standout points and broad statements made on the basis of visual assessment should be avoided**.

We thank the reviewer for highlighting this. Yes, we have designed AIRRscape to encourage analysis via an initial, visual-based overview in order to give the user a broad sense of the repertoire in its entirety, which is then followed up by more granular network topologies. We agree that broad statements based on these initial visual inspections should be qualified. We have reworded the text, which now states “This visual analysis suggested that the overall pattern of heavy chain V-gene family + J-gene pairings versus CDRH3 length distribution appears similar among the CoV-AbDab dataset, the four patient repertoires, and the healthy control repertoire” (new lines 225-227).

The repertoire read count varies widely between samples. The samples with smaller read counts are likely to have thinner vertical bands as a result of the lower numbers. This could be addressed, for example, by providing a sub-sampling option. Otherwise, a note should be made regarding sample size bias.

To clarify this point, heatmaps in AIRRscape group all of the data into bins based on their V+J gene assignments & CDR3 length. These bins are shown as rectangles, whose size is fixed and not dependent on the read count of the bin. However, datasets that are relatively small may have missing rectangles, thus appearing relatively sparse relative to other datasets. Read counts for each bin are provided as a standard feature of AIRRscape, which allow users to note differences in sample sizes.

The SHM metric is used widely in the paper. It is calculated as 100 - v_identity and therefore reflects the level of mutation in the V-REGION rather than overall SHM, which would look also at the level of mutation in the junction, and would therefore require clonal analysis. It would be better to use a different name, perhaps V-SHM, to make this clear.

We thank the reviewer for pointing this out. We confirm that we do not calculate SHM across the entire CDR3 region. We have added text to clarify that ‘SHM’ only applies to the V-gene in our paper (new lines 113-114 & 132-133), in keeping with similar uses of the term SHM in the literature (see Gupta et al. 2020: <https://doi.org/10.3389/fcell.2020.00357> & Lupo et al. 2022: <https://doi.org/10.1371/journal.pcbi.1010167>).

The strength of the tool is in the analysis of convergent sequences, and its utility is usefully shown with reference to the three published studies. **However it is important to note that amino acid sequence convergence at the levels analysed is an imperfect predictor of functional convergence:** sequences will require confirmatory analysis by other means. Because this point is sometimes overlooked, it should be mentioned in the discussion so that users are aware of it.

This is a very important point. We have added text to the discussion to emphasize this (new lines 478-479).

**Other points**

Line 105, 367 – given that clonal analysis is not performed, please explain why changeo-clone needed. Alternatively, if it is needed, why not use the results and incorporate clonal analysis? Do the authors recommend Olmsted over AIRRscape where clonal analysis is available? The comparative merits of the two tools should be set out in greater detail.

Defining clones, and thus changeo-clone, is not needed for AIRRscape visualizations. That being said, users have the option to incorporate changeo-clone, and thus view this clonal information in the AIRRscape tables of selected antibody sequences.

Line 221 – ‘more easily and broadly elicited’ – possibly in the narrow context of mutation steps but this is a sweeping statement.

We acknowledge the reviewer’s critique, and have changed this phrase to ‘therefore may be more easily elicited’ (new lines 236-237).

Line 375 – heavy V-gene *family* (which is a very broad metric).

We have added “as measured by heavy chain V-gene family” for clarification (new lines 416).

Line 389 – This is potentially an important finding, but the dengue sequences are not represented in the covid trees, and the description of these dengue sequences at line 240 is not clear. The reference to 3.3 is not helpful. **Specifically it is important to know whether sequences identical to known functional COVID antibodies were found in the dengue patients** - Otherwise the finding needs to be qualified or supported by more evidence that the sequences are functional against SAVS-CoV-2.

We thank the reviewer for requesting further analysis on this topic. Yes, we agree that it is important to know if SARS-CoV-2 monoclonal antibody sequences are present in dengue patients. We have now included analysis in the results section for comparisons of SARS-CoV-2, HIV-1, & dengue datasets, to examine whether the reported convergent CDR3 motifs are also found across diverse patient repertoires. In this new section we have added one main and 4 supplemental figures (**Fig. 8** & **S10-13 Figs.**). We report that three convergent CDR3 motifs seen in multiple COVID-19 patient repertoires were also found in both HIV-1 and dengue patient repertoires. We have also added the caveat that laboratory experiments are needed to determine whether these sequences are functional against SARS-CoV-2 (new lines 431-433).

----------------------------------------------------------------------------------------------------------------

**Reviewer 2:**

**Major Points**

There is insufficient discussion about previous tools in this space. While there aren't many AIRR-seq visualization tools to compare to, there are some:

●	**"AncesTree**: An interactive immunoglobulin lineage tree visualizer" by Foglierini et al.

●	**Olmsted** by Ralph et al.

●	"**sumrep**: A Summary Statistic Framework for Immune Receptor Repertoire Comparison and Model Validation" by Olson et al.

The above tools don't focus on convergence analysis, like AIRRscape does. Then again, the paper is written as if AIRRscape is a general tool and not one focused on **convergence analysis, an exciting area in need of visualization tools**. **The authors should update the text to make this clear and better position their tool in the space of the AIRR-seq visualization tools.** This would increase its novelty and discoverability.

We thank the reviewer for pointing this out and for highlighting that convergence in antibody responses represents an exciting area of current research in the field. We agree that a more complete overview of the available tools can highlight the important use cases that AIRRscape could provide to the community. We have created a new **Table 1** that enumerates the available tools and their ideal use cases. Our goal with developing AIRRscape was to enable global comparative analyses of immune repertoires in order to identify convergent motifs. We have included new text to highlight the novelty of AIRRscape in its ability to examine convergence (new lines 54-56 & 485-486).

Since the tool is focused on convergence analysis, it should be much easier to identify bins that contain rearrangements from multiple people and/or from people in multiple groups. Data can be faceted by source, then the user has to bounce back and forth trying to identify equivalent bins. Or the combined data could be used but then the user has to go searching for bins with overlapping data. An option should be added to highlight bins with overlapping sequences.

The current version of AIRRscape identifies convergence in a target-dependent manner (i.e. users select antibody sequences of interest and then create topologies to identify convergence at various amino acid identity thresholds). We agree with the reviewer that a target-agnostic approach, which would identify convergence throughout the selected repertoires, is important. We point out in the limitations section of the discussion that while AIRRscape is designed to easily explore these datasets, it does not currently include algorithms that would search for such convergences (new lines 479-482). In the closing statement we invite the community to contribute improvements to the AIRRscape code, including methods to make it easier to identify convergence at a global level (new lines 495-497).

As a practitioner of BCR analysis, **it's not clear from the text how much work is needed to get your own data ready for use in AIRRscape**. The paper does mention AIRR community standards but doesn't describe what other user supplied metadata is needed in order to get the faceted displays shown in the paper. Maybe this is simple but it's not described in the paper, even briefly.

We thank the reviewer for raising this important issue. We have made several changes to the tool and to the manuscript in order to further enable users to load their own data into AIRRscape. On the tool front, our updated version of AIRRscape now implements all of the preprocessing script as part of the *Import Data* tab in the Shiny web interface, making it much easier (and visible) to input any AIRR-seq dataset processed using the AIRR Community standards. On the manuscript front, we have added several explicit references to this new *Import Data* feature (see sections 2.2 and 2.3). Regarding metadata - AIRRscape does not require any metadata per se (as is now noted in section 2.3, new lines 153-155), but rather simply asks for the name of the dataset(s) being uploaded. That name is then transferred to the AIRRscape visualizations.

**The authors commitment to open science, publishing on bioRxiv and making their code fully available on github is to be lauded.**

We thank the reviewer for recognizing the critical importance of open science and open source software.

## **Minor Points**

Some of the author's analysis includes antibodies for which we only know the a.a. sequence. The method they use to reverse translate (using tblastn) is clear. The github repository has some additional details: "search for 100% sequence match to nucleotide sequences". It is not clear how many sequences were reverse translated with 100% a.a. match. Was it all of them? The authors also mention in the github repository that some sequences were "remove… with artificially high SHM due to codon optimization". How many were removed? This suggests that SHM levels for antibodies for which we only know the a.a. sequence should be left as NAs in the data.

We have omitted SHM values for 44 protein sequences from one study (Seydoux et al., 2020; <https://www.biorxiv.org/content/10.1101/2020.05.12.091298v1>), out of 4306 protein sequences in the SARS-CoV-2 mAbs dataset (1.0% omitted). We report SHM values, calculated after the reverse translate step, for 1493 of 4306 protein sequences in the SARS-CoV-2 mAbs dataset and 59 of 98 in the IEDB HIV-1 dataset. For these CoV-AbDab antibodies and HIV antibodies from the IEDB, we have changed the code so that these particular SHM values are now highlighted in blue. Thus, AIRRscape users can immediately note the additional inference. We have also noted this change in the text (new lines 117-118). While we agree with the reviewer that this reverse translation step is imprecise, we think that its inclusion in AIRRscape (now qualified in the text and flagged in blue color in the tool) does offer value, particularly given the wealth of SARS-CoV-2 mAb protein sequences available in Cov-AbDab.

gf_vgene should be used for the V-segment gene family instead of the ambiguous gf.

We agree that gf is ambiguous, and have changed it to the shorter vgf, and similarly changing gene to vgene. We also updated **Figures 1** & **3** to reflect these changes.

I like that the authors emphasize that the phylogenetic trees don't indicate common ancestry. This is a point that is something forgotten or ignored.

The discussion section spends too much time recapitulating the results section. This should be reduced.

We have reduced the length of the discussion section, removing text that was previously stated in the results or methods sections.

----------------------------------------------------------------------------------------------------------------

**Reviewer 3:**

Waltari et al present AIRRscape, an R Shiny tool to visualize and analyze B-cell receptor repertoires. This is performed by collapsing receptor sequences by their V gene, J gene and CDR3 amino acid sequence into a single bin, and calculating the average SHM, max SHM and # of read counts per bin. The authors then show the application of AIRRScape on four different datasets, a COVID-19, HIV-1, Dengue and healthy control datasets. As the authors note, adaptive immune receptor repertoire sequencing **(AIRR-seq) is becoming more extensively used and there is a critical need to develop tools to analyze datasets in both an interactive and static fashion.** This has been critical in other fields such as genomics with the tools IGV and the web browser application ‘UCSC Genome Browser’. While AIRRscape currently has limitations, such as only considering CDR3, as the authors note, AIRRscape represents an initial stage of development for the visualization and interaction of AIRR-seq data.

We thank the reviewer for identifying the critical need to develop tools to interactively visualize AIRR-seq datasets.

**Major revisions:**

1.	It would be good to create a separate **R script containing the `shinyprocess` function that accepts an AIRR community standard file and creates a data file needed by AIRRscape**. While this might defeat the purpose of being user-friendly to people who do not use the command line, the ‘airrscape_preprocessing.R’ file doesn’t seem user-friendly to individuals with less programming experience. At the very least the GitHub page should have instructions on how to visualize a dataset that is not part of the AIRRscape. Instructions should include what lines to edit, lines to run, etc.

We thank the reviewer for raising this important issue. We have made several changes to the tool and to the manuscript in order to further enable users to load their own data into AIRRscape. On the tool front, our updated version of AIRRscape now implements all of the preprocessing script as part of the *Import Data* tab in the Shiny web interface, making it much easier (and visible) to input any AIRR-seq dataset processed using the AIRR Community standards. On the manuscript front, we have added several explicit references to this new *Import Data* feature (see sections 2.2 and 2.3). In addition, for those users that wish to use the command line to preprocess their own data prior to AIRRscape analysis, the preprocessing script remains available on GitHub.

2.	In lines 210-212, the authors state that “This analysis revealed that the overall pattern of heavy chain V- gene + J-gene usage versus CDRH3 length distribution does not differ among the CoV-AbDab dataset, the four patient repertoires, and the healthy control repertoire (Fig 4).” While the patterns across datasets look similar, there are clear differences. There seems to be no SARS-CoV2 mAbs with IGHV6 genes and IGHV2_IGHJ1 and IGHV2_IGHJ2 gene pairs. The Nielsen et al cohort also doesn’t seem to have IGHV5_IGHJ1 or IGHV5_J2 sequences. The phrase “does not differ” in **line 211** may be too strong. It would be good to run statistical test to determine if that is true. **Alternatively, ‘does not differ’ can be loosened up to ‘visually the distributions appear similar’, and the differences can be expanded on.**

We thank the reviewer for highlighting this. Yes, we have designed AIRRscape to encourage analysis via an initial, visual-based overview in order to give the user a broad sense of the repertoire in its entirety, which is then followed up by more granular network topologies. We agree that broad statements based on these initial visual inspections should be qualified. We have reworded the text, which now states “This visual analysis suggested that the overall pattern of heavy chain V-gene family + J-gene pairings versus CDRH3 length distribution appears similar among the CoV-AbDab dataset, the four patient repertoires, and the healthy control repertoire” (new lines 225-227).

3.	**I couldn’t create a topology using the code on GitHub. I kept receiving an ‘Error: argument is of length zero’ message**. I tried on firefox and chrome. I had the rows selected with the same CDR3 length but it kept producing the same message.

We would like to thank the reviewer for identifying this error. This error was not seen in initial testing, however we have recently been able to reproduce it. This error arises after users select from the upper table to make topologies, rather than the lower table. When a user creates a bounding box to select multiple bins, the antibodies are shown in the upper table, rather than the lower table that appears when a single bin is clicked. To better constrain the CDR3 length for downstream topology making, we only have enabled topologies after clicking on a single bin, then selecting sequences of identical CDR3 length from the lower table. We have expanded the ‘Tips’ section of the README documentation to include this error, as well as additional errors that users may encounter.

**Minor revisions:**

1.	Under ‘2.2 Data processing’, can the authors add the parameters and values used for ‘changeo-igblast’? It will also be good to include the allele database used for ‘changeo-igblast’. While AIRRscape is agnostic to igblast parameters and allele databases used, this information should be added for completeness and reproducibility sake.

We used the pre-loaded ‘changeo-igblast’ and ‘changeo-clone’ pipelines in the Immcantation Docker container (v4.1.0). Thus we used the human IG database from igblast v1.16.0. We have added text making it clearer that we are running the Dockerized pipeline (new lines 110-112).

2.	A reference to **VDJbase** is needed. **VDJbase** produces reports and also has interactive modules that lets individuals analyze publicly available AIRR-seq data and determine haplotype, gene and allele usage and other AIRR-seq metrics.

We have now included a reference to VDJbase (in the new **Table 1**).

3.	For table 1, ‘Sample’ presumably means the sample used from the dataset for this study. Can the authors explain why some rows in the table don’t have a ‘Sample’ id.

Most of those blanks are due to the fact that those datasets are a collection of antibodies (in italics). We have added a sentence to the table legend clarifying this point: “Datasets in italics are collections of antibodies”. We did indeed miss the sample id for the healthy control, and we have added it to the table.

4.	For lines 108 to 110, can the authors explain which database was used for tblastn, and why the protein sequences were not converted to nucleotide sequences and aligned to an allele database?

We used the NCBI nr/nt database for the tblastn search and have added this point to the text (new lines 115-117). In those cases where nucleotide sequence is unavailable (see methods section 2.2), the protein sequences are indeed converted to nucleotide sequences using tblastn, followed by allele assignment.

5.	There is a field in AIRR standards that contains the CDR3 sequence. Could this be used instead determining the CDR3 sequence from the junction field?

The *CDR3* field is not always populated. For example, while in our experience datasets downloaded from the OAS database usually include this field, datasets from the iReceptor database do not (instead it is simply blank). In contrast, the *junction_aa* field is populated in all databases we have encountered, and thus we use that field for conversion to the CDR3 sequence.

6.	In line 262-263, the authors state that the V-gene + J-gene usage is similar. However, gene usage is defined either as clonal gene usage or repertoire-wide gene usage and is the frequency of reads (or clones) with a particular gene. Figure 2A doesn’t show gene usage. It shows the SHM rate for different pairs of IGHV+J genes+CDR3 length sequences. **I believe the authors mean the IGHV+J gene pairs are similar.** Line 211 also makes reference to V-gene + J-gene usage. This should be changed to ‘pair’

We thank the reviewer for this critique. The reviewer is correct in that AIRRscape examines assigned V-gene family + J-gene combinations, which isn’t strictly repertoire-wide gene usage. We have changed these two sentences from “V-gene + J-gene usage” to “V-gene family + J-gene pairings” (new lines 225-227 & 275-277).

7.	**R version > 4.1.0 is needed for phangorn,** it would be good to add this minimum version to the GitHub page.

In our hands, we have successfully used AIRRscape with both an older set of R packages using R 4.0.3 and a newer one using R 4.2.0. We have added the versions of R and RStudio used in making AIRRscape and a list of the dependencies to the GitHub repository README. For the updated version of AIRRscape now on the web, the deployment step resolves any R version requirements.

----------------------------------------------------------------------------------------------------------------

**Reviewer 4**

This manuscript describes AIRRscape, an R Shiny application that can be run in the browser, and that allows for the comparison of antibody sequences based on V and J gene, and CDR3 length. It visualizes these antibody characteristics in the form of heatmaps and can examine the convergence of clonotypes by constructing phylogenetic-like trees. The three use cases illustrate potential applications of the tool. The source code is publicly available on GitHub. **With the major comments addressed, especially concerning how it compares to other similar tools, I think AIRRscape could be a useful contribution to the field, particularly for researchers with less programming experience.**

We thank the reviewer for identifying AIRRscape as a potentially useful contribution, in particular for those researchers with less programming experience. A key development goal of AIRRscape is to reduce barriers and to enable users to perform comparative immune repertoire analysis.

**Major comments:**

- Please provide an overview table of what new or improved functionality AIRRscape provides compared to other existing tools for antibody analyses, such as

**VDJtools** <https://doi.org/10.1371/journal.pcbi.1004503>,

**immunarch** <http://doi.org/10.5281/zenodo.3367200>,

**PASA** <https://doi.org/10.1371/journal.pcbi.1008607>.

We agree that a more complete overview of the available tools can highlight the important use cases that AIRRscape could provide to the community. We have created a new **Table 1** that enumerates the available tools (including VDJtools, immunarch, and PASA, among others) and their ideal use cases.

- Please update Figure 1 to show an overview of all types of AIRR analyses that are possible in AIRRscape.

We have updated **Figure 1** to include a bullet point overview of the types of AIRR analyses that are available in AIRRscape.

- How are motifs defined and how are they discovered (e.g., line 64)? In line 203, the authors mention “healthy control motifs” – what does this mean? **Please include the definitions and explanations of motifs in the manuscript.**

We have strived to make AIRRscape agnostic with respect to defining clonal families or motifs. Thus we used the term ‘motif’ in the manuscript to simply refer to a collection of CDR3 sequences. To reduce confusion, we have changed our wording in both of these places, replacing ‘motifs’ with ‘sequences’ (new lines 82 & 216-218).

- In multiple places throughout the manuscript, the authors mention the increasing size of the antibody repertoires and how AIRRscape can help. For example, the author summary (line 27) states: “With AIRRscape, we enable large-scale immune repertoire visualization and analysis...”. **How scalable is it? Please quantify scalability.**

Using a reasonably powerful laptop (16GB RAM, quad-core), we can quickly (within 5 seconds) load heatmaps visualizing datasets of 100,000 - 500,000 sequences. Creating topologies is a more difficult task, and finding the nearest sequences within larger bins of 1,000 - 5,000 sequences takes between 1 and 5 minutes in our hands. We have included these general guidelines in the GitHub documentation.

**Minor comments:**

- **R and R studio versions are missing, along with dependencies**. It would be nice to have all that resolved automatically if possible or to have a list of required packages and tools with versions. With this resolved, AIRRscape worked well locally for me.

In our hands, we have successfully used AIRRscape with both an older set of R packages using R 4.0.3 and a newer one using R 4.2.0. We have added the versions of R and RStudio used in making AIRRscape and a list of the dependencies to the GitHub repository README. For the updated version of AIRRscape now on the web, the deployment step resolves any R version requirements.

- The authors mention “processed flat files” multiple times (e.g., line 142): **what does flat refer to in this context?**

We are referring to a simple tab- (or comma-) delimited file, but understand the term may be confusing. We have updated the text changing ‘flat file’ to ‘tab-delimited file’ (new lines 108 & 151-153).

- In line 172, it says that users may choose to construct the topology via either neighbor-joining or parsimony tree building methods. Is this performed using some package or is it a native AIRRscape implementation?

The tree building is indeed using an R package - phangorn to be precise. We have added the reference for phangorn to this sentence (new line 188).
